# Antibacterial efficacy of combined atmospheric cold plasma and hydrogen peroxide treatment on a wound surrogate

**DOI:** 10.1016/j.bbrep.2025.102296

**Published:** 2025-10-03

**Authors:** Praj K. Patel, Preisha Mishra, Habiba K. Ashour, Neil R. Mandar, Safa Mbarki, Yong Mao, Suneel Kumar, Francois Berthiaume, Aaron D. Mazzeo

**Affiliations:** a Department of Mechanical and Aerospace Engineering, Rutgers University, 98 Brett Road, Piscataway, NJ, 08854, USA; b Department of Biomedical Engineering, Rutgers University, 599 Taylor Road, Piscataway, NJ, 08854, USA; c Laboratory for Biomaterials Research, Department of Chemical and Chemical Biology, 145 Bevier Road, Rutgers University, Piscataway, NJ, 08854, USA

## Abstract

This study aims to understand the potential combined effects of treating wound-like tissue surfaces with cold plasma (CP) and hydrogen peroxide. We assess how CP treatment generated by a surface dielectric barrier discharge (SDBD) device achieves bacterial inactivation on two test surfaces: agar plates, representing a surface with uniform topology, and muscle tissue from a thin-sliced chicken breast, representing a non-uniform topology mimicking a wound-like surface. A 10-min CP treatment inactivates *Escherichia coli (E. coli)* with up to 7 log reduction in colony-forming units (CFU) on a smooth agar surface; however, on chicken breast, the same treatment yields a 0.88 log reduction. By comparison, the common antiseptic H_2_O_2_ (3 %) yields a 1.06 log CFU reduction on chicken breast after 10 min of treatment. Simultaneous treatment with CP and H_2_O_2_ increases *E. coli* inactivation to 1.69 log CFU. Bacterial inactivation is less efficient on the chicken tissue than on smooth agar surfaces. Furthermore, the CP-H_2_O_2_ combination significantly improves bacterial inactivation, which can be further enhanced by extending treatment time. This work demonstrates an approach to evaluating the efficacy of combining CP with liquid antimicrobial treatments on an accessible wound surrogate with complex morphology and biochemistry. This approach has the potential to serve as a fast method to screen candidate treatments before performing animal studies.

## Highlights

• Cold plasma (CP) and hydrogen peroxide exhibited bactericidal properties independently.
• Bacterial reduction on tissue proved more difficult than inactivation on an agar surface.
• A 10-min treatment of CP and hydrogen peroxide, in combination, demonstrated an increase in bacterial inactivation.

## 1 Introduction

Open skin wounds are prone to bacterial colonization, which can lead to delayed healing, amputation, and sepsis [1,2]. A challenge in wound care is the formation of bacterial biofilms, which are resistant to both antimicrobial treatments and immune system responses [3,4].

Current methods for bacterial control include cleaning wounds using isotonic, hypotonic, surfactant antimicrobial, and antiseptic solutions [5]. These treatments work by disrupting cellular metabolism [6], but their efficacy can vary depending on wound characteristics, such as wound size, texture, and presence of foreign material [7]. Moreover, antibiotic resistance has reduced the efficacy of these treatments [8], with studies showing up to 70 % of wound infection-causing bacteria are resistant to at least one antibiotic [9]. New methodologies for bacterial inactivation are required to address these limitations. Atmospheric dielectric barrier discharge cold plasma (ADCP), an alternative anti-bacterial treatment, uses cold plasma (CP) formed by ionizing air with time-varying potentials and electrodes. A device typically consists of at least one electrode covered in an insulating dielectric [10]. While CP is often generated in a volume of space, surface CP devices produce plasma off a surface/edge and can operate with lower applied voltages than those used with volume-based devices. To create surface CP, surface dielectric barrier discharge (SDBD) devices may use asymmetric electrodes with CP forming on the edges of one of the electrodes [11]. CP contains reactive oxygen and nitrogen species (RONS), which can then oxidize organic material, thus sterilizing surfaces while producing O, O_2_^−^, OH^−^, OH, H_2_O_2_, N, N_2_∗, and NO^−^ [12]. Flexible surface generators may be appropriate for wound sterilization, where surfaces are not flat and have difficult-to-reach crevices in which microbes can collect [13].

Surface topology appears to influence CP efficacy. For example, ADCP treatment with a plasma jet (492 s, ±295 V) on apple and cantaloupe peels led to more inactivation of *Enterobacter aerogenes* (∼1.86 vs. ∼0.61 log reduction) on the smooth apple surface than on the rough cantaloupe peels [14]. To increase bacterial killing, efforts have supplemented CP with other treatments, which have yielded higher bacterial inactivation. Examples of these combined treatments include (1) CP plus an antimicrobial CaO solution, which achieved a higher *Penicillium digitatum* killing on mandarins than individual treatments of either CP or CaO [15], (2) citric acid combined with CP to inactivate *Escherichia coli (E. coli)* and *Listeria monocytogenes* in coconut water [16]. Some studies have even shown a synergistic interaction between CP and other treatments, achieving higher bacterial killing than the sum of the two individual treatments. Examples include (1) CP plus organic acids (e.g., lactic and gallic acid) to inactivate *Salmonella typhimurium* on poultry meat surfaces [17], (2) ethanol combined with CP to inactivate bacteria in bee pollen [18], and (3) hydrogen peroxide combined with CP to inactivate *Salmonella Typhimurium* on cabbage slices [19].

Hydrogen peroxide (H_2_O_2_) on its own is a common disinfectant in wound care for its ability to oxidize a variety of materials, primarily through the generation of hydroxy radicals (·OH) [20]. For example, 3 % (v/v) H_2_O_2_ has been shown to reduce *S. epidermidis* in biofilms by 0.37 log after 1 min of treatment [21]. Furthermore, CP in combination with H_2_O_2_ has shown a significant increase in the concentration of ·OH radicals, which facilitates the penetration of nitrogen-based species through cellular membranes to damage proteins and facilitate cell death [19]. An example of synergistic disinfection was a combined treatment of 3 % (v/v) H_2_O_2_ and DBD-based CP applied for 90 s, which inactivated 6-log bacteria on an agar surface, about 3-log and 2-log more inactivation than individual treatments of H_2_O_2_ and CP [22].

In this study, we investigated the potential for CP, used alone and in combination with hydrogen peroxide (H_2_O_2_), to inactivate bacteria on animal tissue surfaces (chicken breast) used as wound surrogates. Bacterial killing on chicken muscle – used as a surrogate for the wound bed – was much less efficient than on a smooth agar surface used as a control. Nonetheless, a combination of CP and hydrogen peroxide treatments significantly increased bacterial killing.

## 2 Materials and methods

### 2.1 Preparation of chicken samples

The chicken breast (Perdue’s thin-sliced chicken with a thickness of 3–4 mm), purchased from a local grocery store, was used. A surgical knife cut the chicken breast into 60-mm diameter circles, each placed in a 60-mm diameter Petri dish. To check for any bacteria present in the chicken, we used a biopsy punch (10-mm diameter) to cut a small piece of the chicken from the same batch. The cut piece, mixed in a vortex mixer for 30 s in a 5-mL tube containing 1 mL of PBS. From this solution, 100 μL was spread onto an agar plate, which was incubated for 24 h at 37 °C. After the incubation period, the plate appeared clear, indicating no bacterial growth.

### 2.2 Preparation of *E. coli*

The agarose solution was prepared by dissolving 20 g of Difco Tryptic Soy Agar powder in 500 mL of deionized water. The agar solution was autoclaved (Ward’s BioClave) at 121 °C for 15 min. Then, we dispensed 20 mL of agar solution per 100-mm diameter Petri dish (VWR) under sterile conditions. Dishes were allowed to cool to room temperature. We used a nichrome loop to inoculate *E. coli* (ATCC 700928) on the agar plates and placed them in an incubator (Quincy Lab Incubator, 12-140E) for 24 h at 37 °C. We then transferred one colony of *E. coli* to a dual-position culture tube with 1 mL of Difco Tryptic Soy Broth (TSB) and incubated these tubes at 37 °C while shaking (125 rpm) for 24 h in an incubator (Lab Companion SI-300). After incubation, we diluted the colonized broth with TSB at a ratio of 1:3 to achieve an acceptable optical density that can be measured in a spectrophotometer. Optical density (ThermoScientific, Nicolet Evolution 100 spectrophotometer) was measured using 1 mL of the bacterial solution in a 1.5-mL disposable cuvette and converted to a bacterial concentration in CFU/mL. Once the bacterial count was known through the spectrophotometer, the *E. coli* suspension was then centrifuged (8000 RCF) for 5 min. Finally, the pellet was suspended in PBS solution to obtain a 10^8^ CFU/mL concentration.

### 2.3 SDBD device fabrication and cold plasma generation

**Fig. 1:**
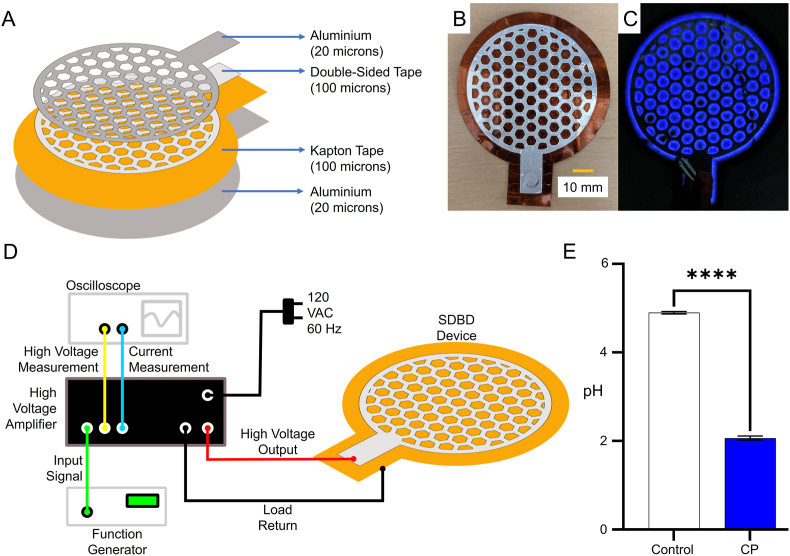
Schematic of SDBD device and effect of CP on pH level. (a) An exploded view of the SDBD device. (b) Image of the SDBD device without applied potential. (c) SDBD device with applied potential displaying a blueish glow due to the production of UV/low wavelength visible light. (d) CP system, including a function generator, amplifier, oscilloscope, and SBDB device. (e) Graph showing pH values before and after 10 min of CP treatment to confirm production of acidifying RONS species. Data are presented as mean ± standard error of the mean (n = 4/group). Data was analyzed using a Student's t-test. ∗∗∗∗p < 0.0001.

The SDBD device consists of a 100-micron-thick tape (Tape Master Kapton Polyimide) between two 20-μm aluminium foil sheets. We used 100-μm double-sided adhesive tape (Elizabeth Craft Designs) to attach the top electrode and dielectric. A honeycomb-like array of hexagons was cut from the top electrode (Fig. 1A); the hexagonal shapes allow plasma generation on their edges. A GW Instek SFG-2104 Synthesized Function Generator provided a sinusoidal signal with a frequency of 2.5 kHz and amplitude of ±2.5 V to a Trek Model 10/10 B-HS Amplifier, which amplified the signal to ±2.5 kV to drive the electrodes (Fig. 1D). A Tektronix TDS 3032 Oscilloscope connected to the amplifier monitored voltage and current in real-time. The SDBD device without any applied potential is shown in its inactive state (Fig. 1B), while plasma generation along the hexagonal edges under an applied potential is visible during operation (Fig. 1C).

### 2.4 Inoculation and CP treatment

We uniformly spread 100 μL of 10^8^ CFU/mL solution on the test surface (agar or chicken breast) with L-shaped (Fisherbrand) cell spreaders. To verify the bacterial load applied to the surfaces, we took 100 μL of the 10^8^ CFU/mL suspension, transferred it into 900 μL of PBS, and performed five consecutive 1:10 serial dilutions. We then plated 100 μL of the final dilution onto agar and incubated the plates at 37 °C for 24 h. After incubation, we counted colonies to calculate the total CFU present on the test surfaces. For CP treatment, we exposed the inoculated agar or chicken surface to plasma for 10 or 20 min. For hydrogen peroxide treatment, we spread 0.5 mL of 3 % H_2_O_2_ (CVS Health) solution across the inoculated chicken surface and waited 10 min before sample collection for CFU quantification. For combined CP-H_2_O_2_ treatment, we spread 0.5 mL of 3 % H_2_O_2_ over the inoculated chicken surface immediately, followed by 10 or 20 min of CP treatment.

### 2.5 Sample collection for CFU quantification

We collected samples from five groups: control (no treatment), CP-treated, H_2_O_2_-treated, and CP–H_2_O_2_–treated for 10 and 20 min. We used an Acuderm 10-mm biopsy punch to collect four pieces from a treated chicken breast sample, placing each piece in polypropylene centrifuge tubes (ThermoScientific) with 1 mL of PBS solution. The tubes were vortexed for 30 s. The resulting suspension was diluted 1:10 three times, and 100 μL of the final dilution was applied to 100-mm agar plates. After incubation for 24 h at 37 °C, we determined the bacterial colony count.

### 2.6 Determination of pH change of the inoculum solution and chicken surface pre- and post-CP treatment

We suspended 0.5 mL of H_2_O_2_ and 0.1 mL of PBS in a 60-mm Petri dish and subjected it to CP treatment for 10 min. This experimentally measured pH level (Orion VersaStar Advanced Electrochemistry Meter, ThermoScientific) of the solution occurred before and after CP treatment to verify that the setup generated acidic species and to confirm effective generation of CP. In addition, we performed another experiment in which the inoculum solution (0.5 mL of H_2_O_2_ and 0.1 mL of PBS) was suspended directly on the surface of the cut chicken placed in a 60-mm Petri dish, as shown in Fig. 3, and subjected to CP treatment for 10 min.

## 3 Results

### 3.1 Bacterial inactivation on agar plates

**Fig. 2:**
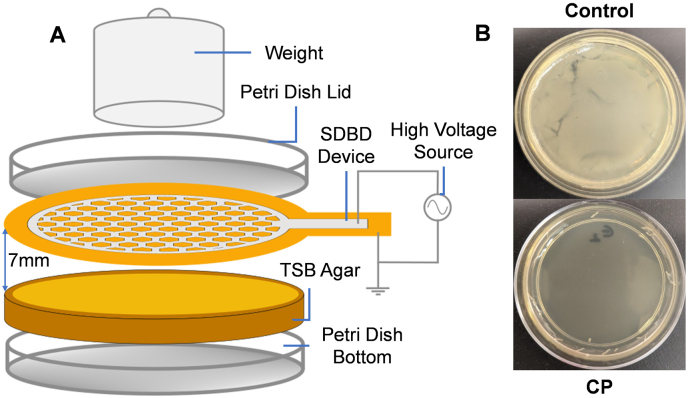
Effect of cold plasma (CP) on bacterial inactivation using agar surface. (a) Schematic of CP treatment setup of agar plates and (b) Agar plates after 24 h without (Control) and with plasma treatment (CP). Number of samples n = 4 for the control and n = 4 for the CP results.

To measure the effectiveness of a single CP treatment against *E. coli* bacteria on an agar surface, approximately 10^7^ CFU (7.746 ± 0.075 log CFU, four samples) were inoculated over the entire surface of the agar. A 10-min CP treatment was applied (Fig. 2A), after which the plate was incubated for 24 h. No bacterial colonies formed (Fig. 2B, see bottom image) in plasma-treated agar plates, suggesting that the entire bacterial inoculum was killed, thus corresponding to a 7-log CFU reduction. In contrast, similarly inoculated and incubated agar plates without plasma treatment (Fig. 2B, see top image) yielded a bacterial lawn covering the entire plate surface with a turbid appearance.

**Fig. 3:**
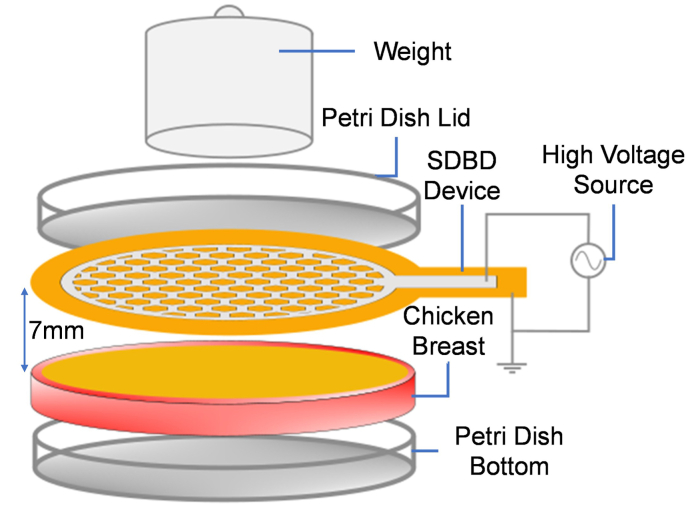
Experimental set-up for bacterial inactivation on the chicken surface. Schematic of cold plasma treatment on the chicken surface for variable time points alone or in combination with H_2_O_2_.

### 3.2 Bacterial inactivation on chicken breast using plasma treatment

**Fig. 4:**
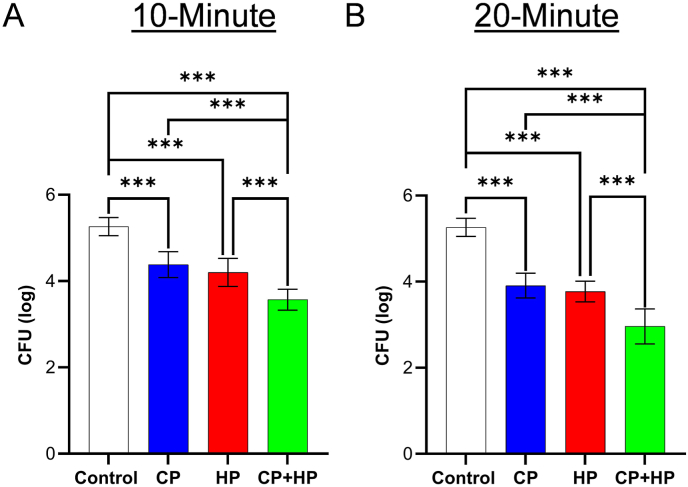
Bacterial inactivation on chicken surfaces using cold plasma (CP) alone, hydrogen peroxide (HP) alone, and a combination of cold plasma (CP) and hydrogen peroxide (HP). Data are presented as mean ± SD (log CFU; n = 8–12). Data were analyzed using one-way ANOVA followed by post-hoc Tukey’s HSD test. ∗∗∗p < 0.001.

We measured bacterial inactivation on the chicken surface (Fig. 3) as a wound bed surrogate similar to agar experiments, as mentioned in Fig. 2. A 10-min CP treatment alone reduced the bacteria significantly by 0.88 log CFU (p < 0.001). A 10-min 3 % v/v H_2_O_2_ treatment alone reduced bacteria by 1.06 log CFU (p < 0.001). When the two treatments were done simultaneously, we found a reduction of 1.69 log CFU (p < 0.001; Fig. 4), which exceeded the reductions observed from the individual treatments.

We also increased the CP treatment time to 20 min, which resulted in a 1.35 log CFU reduction (p < 0.001), which was 0.47 log more than the 10-min CP treatment. A 20-min H_2_O_2_ treatment achieved 1.49 log CFU inactivation, which was 0.43 log more than the 10-min H_2_O_2_ treatment. The 20-min combined CP-H_2_O_2_ treatment achieved a 2.30 log CFU reduction, which was 0.61 log higher than the 10-min combined treatment (Fig. 4). This suggests the simultaneous 10- and 20-min treatments with CP and H_2_O_2_ achieved higher bacterial inactivation than the individual treatments.

### 3.3 Change in pH of the inoculum solution and chicken surface pre- and post-CP treatment

A 10-min CP treatment lowered the pH level of the inoculum from ∼5 (4.9 ± 0.05, n = 4) to ∼2 (2.06 ± 0.08, n = 4) after plasma treatment (Fig. 1E), increasing the acidity of the solution. When the inoculum solution was instead suspended on the chicken surface, the pH of the solution exposed to the chicken was higher – a value of ∼6.6 (6.56 ± 0.055) -than the inoculum that had not been in contact with the chicken. After 10 min of exposure to CP, the inoculum in contact with the chicken had a measured pH of ∼6.1 (6.11 ± 0.004, n = 2).

## 4 Discussion

CP treatments are an emerging approach to decrease the bacterial load on skin wounds. However, there is still an opportunity to understand the effect of the wound environment on the efficiency of bacterial inactivation. In this study, we showed that CP treatment works effectively on a uniform surface (agar) but far less effectively on biological tissue (chicken breast). Similarly, a prior investigation with ADCP reported a ∼5.7 and ∼4.5 log CFU reduction of *E. coli* on stainless steel and rubber, respectively, but could only achieve a ∼1.8 log CFU reduction on pig skin for a 300-s treatment [23].

Taken together, these data clearly suggest that inactivating bacteria on tissue is less efficient than on smooth nonbiological surfaces. Nevertheless, we also show that it is possible to improve bacterial killing significantly on tissue by combining the CP treatment with the H_2_O_2_ treatment. We show that CP and H_2_O_2_ each reduce *E. coli* on chicken breast when used alone, but their combination is more effective. Extending the treatment time makes the combination of CP and H_2_O_2_ even more effective, with the 20-min combined CP-H_2_O_2_ treatment achieving the highest reduction.

In 2009, Canadian Blood Services implemented a new skin disinfection method, which involved a swab stick containing 70 % isopropyl alcohol and 2 % chlorhexidine, which was capable of thoroughly disinfecting 87.5 % of all the treated samples, each having more than 100 bacterial colonies before the treatment. This treatment was reported to have a 0.04 % skin reaction out of 17,500 blood donors who were disinfected per week [24]. The CP-H_2_O_2_ achieves a significantly higher log reduction compared to this CBS method, suggesting its potential for skin disinfection. There are multiple uses of CP for antibacterial treatments [25]. Therefore, the use of H_2_O_2_ in addition to CP – as opposed to CP alone – might be a consideration for future clinical treatments. The combined treatment may be appropriate in cases when biofilms have formed or antimicrobial resistance to common antibiotics is a concern. Combined treatment might also help to reduce CP treatment duration, thus minimizing potential side effects of CP, such as tissue damage or irritation.

The resulting decreased bacterial killing efficiency on tissue versus smooth agar would benefit from further analysis. Surface properties like charge density, wettability, roughness, topography, and stiffness can influence the bacterial binding process to a surface, which affects the effectiveness of inactivation [26]. CP treatment also introduces RONS, typically nitrogen oxides, which have been shown to produce nitric and nitrous acid, which in turn lowers the surface pH [27]. We show in this study that CP treatment lowered the pH of the inoculum solution (0.5 mL H_2_O_2_ and 100 μL of PBS), confirming that our CP setup generates acidic species in liquids.

A wound environment with a pH of 6 or above is a prerequisite for the growth of most human-relevant pathogens [[28], [29], [30]]. Lowered pH may correlate with higher bacterial killing, although the tendency toward higher acidity may be mitigated by the alkaline milieu of wounds (pH 7.5–8.9) [28]. We observed a similar effect in our study, where the pH of the inoculum solution suspended on chicken tissue decreased only slightly (from pH ∼6.5 to ∼6.1) following CP treatment, indicating that the tissue environment buffers against changes in pH.

In addition, biological surfaces contain large amounts of soluble and insoluble proteins, as well as nucleic acids, which are susceptible to RONS oxidation [31] and, therefore, can scavenge some of the RONS away from the bacterial targets. It is plausible that the H_2_O_2_ may react with these oxidizable moieties, thus helping CP-derived RONS reach the bacteria when the CP-H_2_O_2_ combined treatment is used. Further studies could explore these mechanisms in living animal models.

In conclusion, this study demonstrates the potential of using CP in combination with H_2_O_2_ for disinfecting tissue-like surfaces. Testing parameters, such as low temperature, atmospheric pressure, and easy reproducibility, underline extending the study to contaminated human skin. The positive antimicrobial effects may also influence future studies combining other cleaning agents with SDBDs to disinfect wound-like surfaces.

## CRediT authorship contribution statement

**Praj K. Patel:** Writing – review & editing, Writing – original draft, Visualization, Validation, Supervision, Software, Resources, Project administration, Methodology, Investigation, Formal analysis, Data curation, Conceptualization. **Preisha Mishra:** Writing – original draft, Methodology, Investigation. **Habiba K. Ashour:** Methodology, Investigation. **Neil R. Mandar:** Writing – original draft. **Safa Mbarki:** Methodology, Investigation. **Yong Mao:** Writing – review & editing, Supervision, Resources, Methodology. **Suneel Kumar:** Writing – review & editing, Visualization, Validation, Supervision, Software, Resources, Methodology, Investigation, Formal analysis, Data curation. **Francois Berthiaume:** Writing – review & editing, Visualization, Validation, Supervision, Resources, Methodology, Investigation, Formal analysis. **Aaron D. Mazzeo:** Writing – review & editing, Writing – original draft, Visualization, Validation, Supervision, Software, Resources, Project administration, Methodology, Investigation, Funding acquisition, Formal analysis, Data curation, Conceptualization.

## Declaration of competing interest

The authors declare the following financial interests/personal relationships which may be considered as potential competing interests:Aaron Mazzeo has patent #US20200246496A1 issued to Rutgers University. Aaron Mazzeo has patent #US20170224856A1 issued to Rutgers University. If there are other authors, they declare that they have no known competing financial interests or personal relationships that could have appeared to influence the work reported in this paper.

## Data Availability

Data will be made available on request.

## References

[1] Browne A.C., Vearncombe M., Sibbald R.G. (2001). High bacterial load in asymptomatic diabetic patients with neurotrophic ulcers retards wound healing after application of dermagraft. Ostomy/Wound Manag..

[2] Hogan B.K. (May 2012). Correlation of American burn association sepsis criteria with the presence of bacteremia in burned patients admitted to the intensive care unit. J. Burn Care Res..

[3] Roy S. (2014). Mixed‐species biofilm compromises wound healing by disrupting epidermal barrier function. J. Pathol..

[4] Roy S. (2020). Staphylococcus aureus biofilm infection compromises wound healing by causing deficiencies in granulation tissue collagen. Ann. Surg..

[5] Weir D., Swanson T. (2019). Ten top tips: wound cleansing. Wounds Int..

[6] Reygaert W.C. (2018). An overview of the antimicrobial resistance mechanisms of bacteria. AIMS Microbiol..

[7] Yousefian F. (2023). Antimicrobial wound dressings: a concise review for clinicians. Antibiotics.

[8] Filius P.M.G., Gyssens I.C. (2002). Impact of increasing antimicrobial resistance on wound management. Am. J. Clin. Dermatol..

[9] Ranghar S., Sirohi P., Verma P., Agarwal V. (2014). Nanoparticle-based drug delivery systems: promising approaches against infections. Braz. Arch. Biol. Technol..

[10] Xu X. (2001). Dielectric barrier discharge—properties and applications. Thin Solid Films.

[11] Trosan D., Walther P., McLaughlin S., Salvi D., Mazzeo A., Stapelmann K. (2024). Analysis of the effects of complex electrode geometries on the energy deposition and temporally and spatially averaged electric field measurements of surface dielectric barrier discharges. Plasma Process. Polym..

[12] Bourke P., Ziuzina D., Han L., Cullen P.J., Gilmore B.F. (Aug. 2017). Microbiological interactions with cold plasma. J. Appl. Microbiol..

[13] Xie J., Chen Q., Suresh P., Roy S., White J.F., Mazzeo A.D. (2017). Paper-based plasma sanitizers. Proc. Natl. Acad. Sci..

[14] Bhide S., Salvi D., Schaffner D.W., Karwe M.V. (Aug. 2017). Effect of surface roughness in model and fresh fruit systems on microbial inactivation efficacy of cold atmospheric pressure plasma. J. Food Protect..

[15] Bang I.H., Lee E.S., Lee H.S., Min S.C. (Apr. 2020). Microbial decontamination system combining antimicrobial solution washing and atmospheric dielectric barrier discharge cold plasma treatment for preservation of mandarins. Postharvest Biol. Technol..

[16] Mahnot N.K., Mahanta C.L., Farkas B.E., Keener K.M., Misra N.N. (Dec. 2019). Atmospheric cold plasma inactivation of *Escherichia coli* and *Listeria monocytogenes* in tender coconut water: inoculation and accelerated shelf-life studies. Food Control.

[17] Yadav B., Roopesh M.S. (June 2022). Synergistically enhanced *Salmonella* typhimurium reduction by sequential treatment of organic acids and atmospheric cold plasma and the mechanism study. Food Microbiol..

[18] Chen M., Yang X., Ji Z., Zhao H., Cheng N., Cao W. (Feb. 2024). Combined treatment of drying, ethanol, and cold plasma for bee pollen: effects on microbial inactivation and quality attributes. Food Biosci..

[19] Kim Y.E., Min S.C. (Dec. 2021). Inactivation of *Salmonella* in ready-to-eat cabbage slices packaged in a plastic container using an integrated in-package treatment of hydrogen peroxide and cold plasma. Food Control.

[20] McDonnell G., Russell A.D. (Jan. 1999). Antiseptics and disinfectants: activity, action, and resistance. Clin. Microbiol. Rev..

[21] Presterl E. (Aug. 2007). Effects of alcohols, povidone-iodine and hydrogen peroxide on biofilms of Staphylococcus epidermidis. J. Antimicrob. Chemother..

[22] Gershman S., Harreguy M.B., Yatom S., Raitses Y., Efthimion P., Haspel G. (2021). A low power flexible dielectric barrier discharge disinfects surfaces and improves the action of hydrogen peroxide. Sci. Rep..

[23] Pavlovich M.J., Chen Z., Sakiyama Y., Clark D.S., Graves D.B. (2013). Effect of discharge parameters and surface characteristics on ambient-gas plasma disinfection. Plasma Process. Polym..

[24] Ramirez‐Arcos S., Goldman M. (2010). Skin disinfection methods: prospective evaluation and postimplementation results. Transfusion (Paris).

[25] Apelqvist J. (2024). Cold plasma: an emerging technology for clinical use in wound healing. J. Wound Manag..

[26] Zheng S. (2021). Implication of surface properties, bacterial motility, and hydrodynamic conditions on bacterial surface sensing and their initial adhesion. Front. Bioeng. Biotechnol..

[27] Nyang’au J.O., Sørensen P., Møller H.B. (2024). Effects of plasma treatment of digestates on pH, nitrification and nitrogen turnover during storage and after soil application. Environ. Technol. Innov..

[28] Schneider L.A., Korber A., Grabbe S., Dissemond J. (2007). Influence of pH on wound-healing: a new perspective for wound-therapy?. Arch. Dermatol. Res..

[29] Stewart C.M., Cole M.B., Legan J.D., Slade L., Vandeven M.H., Schaffner D.W. (2002). Staphylococcus aureus growth boundaries: moving towards mechanistic predictive models based on solute-specific effects. Appl. Environ. Microbiol..

[30] Thomas L.V., Wimpenny J.W., Davis J.G. (1993). Effect of three preservatives on the growth of Bacillus cereus, vero cytotoxigenic Escherichia coli and Staphylococcus aureus, on plates with gradients of pH and sodium chloride concentration. Int. J. Food Microbiol..

[31] Valverde M., Lozano-Salgado J., Fortini P., Rodriguez-Sastre M.A., Rojas E., Dogliotti E. (2018). Hydrogen peroxide‐induced DNA damage and repair through the differentiation of human adipose‐derived mesenchymal stem cells. Stem Cell. Int..

